# Psychometric evaluation of the *Intersectional Discrimination Index* for use in Brazil

**DOI:** 10.1590/0102-311XEN009724

**Published:** 2024-09-23

**Authors:** Natália Peixoto Pereira, Carolina Saraiva de Macedo Lisboa, João Luiz Bastos

**Affiliations:** 1 Pontifícia Universidade Católica do Rio Grande do Sul, Porto Alegre, Brasil.; 2 Simon Fraser University, Burnaby, Canada.

**Keywords:** Intersectional Framework, Perceived Discrimination, Psychometrics, Statistical Factor Analysis, Marco Interseccional, Discriminación Percibida, Psicometría, Análisis Factorial

## Abstract

This cross-sectional study evaluated the configural and metric structures of the *Intersectional Discrimination Index* (InDI), an instrument that measures anticipated (InDI-A), dat-to-day (InDI-D), and major (InDI-M) discrimination. Data from a broader study, focused on the impacts of discrimination on the mental health of women living in Brazil, were used. Approximately 1,000 women, selected according to a convenience sampling scheme, answered the InDI and questions about sociodemographic characteristics in an electronic form that was administered in 2021. Exploratory factor analyses and exploratory structural equation modeling were applied to the first half of the sample; for the second, confirmatory factor analysis was conducted. Taken together, the findings suggest that each of the three measures is one-dimensional. However, unlike the study that originally proposed the InDI for use in Canada and the United States, we observed the presence of residual correlations in the three subscales evaluated, all of which were suggestive of content redundancy between specific pairs of items. The three measures showed moderate to strong factor loadings and acceptable fit to the data. InDI exhibited reasonable internal validity, potentially becoming a valuable instrument for investigating the health effects of intersectional discrimination in Brazil. Future studies should evaluate the consistency of these findings, examine the scalar structure of the instrument, and analyze its invariance among different marginalized groups.

## Resumen

Este estudio transversal evaluó las estructuras configural y métrica del *Intersectional Discrimination Index* (InDI), un instrumento que mide la discriminación anticipada (InDI-A), diaria (InDI-D) y mayor (InDI-M). Se utilizaron datos de una encuesta más amplia, centrada en los impactos de la discriminación en la salud mental de las mujeres que viven en Brasil. Aproximadamente 1.000 mujeres, seleccionadas por conveniencia, respondieron el InDI y preguntas sobre características sociodemográficas en formulario electrónico, aplicado en el 2021. Mientras que en la primera mitad de la muestra se realizaron análisis factoriales exploratorios y se realizó un modelado por ecuaciones estructurales exploratorias, en la segunda se realizó un análisis factorial confirmatorio. En conjunto, los hallazgos sugieren que cada una de las tres medidas es unidimensional. Sin embargo, a diferencia del estudio que originalmente propuso el InDI para su uso en Canadá y Estados Unidos, observamos la presencia de correlaciones residuales en las tres subescalas evaluadas, todas ellas sugestivas de redundancia de contenido entre pares específicos de ítems. Las tres medidas presentaron cargas factoriales de moderadas a fuertes e índices de ajuste aceptables. El InDI exhibió indicadores de validez interna razonables, convirtiéndose potencialmente en un instrumento valioso para investigar los efectos de la discriminación interseccional para la salud en Brasil. Los estudios futuros deben evaluar la consistencia de estos hallazgos, examinar la estructura escalar del instrumento y analizar su invariancia entre diferentes grupos marginados.

## Introduction

Discrimination refers to a process by which members of a socially defined group are treated differently and unfairly, simply due to their belonging to that segment of the population ^1^. Since the 1990s, a growing body of research has established the relationships between discrimination and adverse health conditions and inequities ^2^. The majority of this literature concentrates on the health-damaging effects from ethno-racial discrimination, as well as evidence regarding the health impacts from discrimination based on gender, sexual orientation, disability, mental health, and body size ^3^ ^,^ ^4^ ^,^ ^5^ ^,^ ^6^. In addition to research on discrimination stemming from multiple social statuses and positions ^4^, there is growing interest in the health effects of multiple forms of discrimination, and how these various forms intersect ^7^.

Understanding the role discrimination plays in producing and maintaining health inequities represents an important area of research, as unfair treatment may be reduced or completely eliminated from social interactions ^8^. However, the experiences of groups lying at the intersections of race, gender, class, and other markers of social injustice still need to be fully addressed ^9^. Intersectionality has been used to deal with these issues, as it emphasizes the relations between power, systems of oppression, identity, and processes of marginalization ^10^ ^,^ ^11^, with recent application in specific areas of scientific knowledge, including public health ^8^ ^,^ ^10^ ^,^ ^12^.

The relations between systems of oppression have been conceptualized and discussed by U.S. black feminists for a long time ^13^, even before the term intersectionality was coined by Kimberlé Crenshaw ^14^. Such a concept emerged to describe the experiences of African-American women at the end of the 1980s. Intersectionality studies focus on groups experiencing multiple forms of discrimination, which cannot be considered in isolation from each other ^8^ ^,^ ^10^. Even though the number of studies adopting an intersectional perspective has increased considerably over the past few years ^15^ ^,^ ^16^, Public Health scholars still need to tackle some fundamental challenges, such as the need to: (1) develop instruments that assess of intersectional discrimination; (2) clarify the mechanisms underlying health inequities; and (3) investigate groups characterized by other markers of social injustice, in addition to race, gender, and sexual orientation.

Recent investigations have sought to address these challenges through the development of instruments that measure experiences with intersectional discrimination. One of these is the *Intersectional Discrimination Index* (InDI), originally proposed in Canada and the United States by Scheim & Bauer in 2019 ^7^. The InDI is a self-reporting instrument, with 31 items distributed in three subscales that measure anticipated (InDI-A), day-to-day (InDI-D), and major discrimination (InDI-M). To focus on discrimination without any specific attribution, the InDI asks about unfair treatment “because of who you are”. The following description is offered to respondents: “These questions are about experiences related to who you are. This includes both how you describe yourself and how others might describe you. For example, your skin color, ancestry, nationality, religion, gender, sexuality, age, weight, disability or mental health issue, and income”.

The InDI-A consists of nine items evaluated on a 5-point Likert scale, ranging from 0 (i.e., completely disagree) to 4 (i.e., completely agree). This subscale is based on minority stress theories, which argue that repeated exposure to discrimination prompts individuals to be constantly on alert, anticipating these types of experiences ^7^ ^,^ ^17^. The InDI-D, on the other hand, has nine items that are evaluated on a 4-point frequency scale: never; yes, but not in the last year; yes, once in the last year; and yes, many times in the past year. These items were derived from measures that focus on microaggression (e.g., minor insults and invalidations), ableism, fat-shaming, racism, LGBTphobia, and intersectional discrimination, among others ^7^. Finally, the InDI-M is made up of 13 items, which are evaluated on a frequency scale with the following response options: never; once; and more than once. Considering that item nine of InDI-M specifically refers to repeated assault, the question is presented with these response options: no; yes, in one place; and yes, in more than one place. The InDI-M also records whether discriminatory experiences occurred within the past 12 months are also analyzed. Major discrimination items were adapted from other scales ^18^, and refer to extreme situations, such as denial of service, property damage, and physical violence.

As a means to document specific forms of discrimination, the InDI additionally offers respondents an opportunity to attribute their experiences with discrimination to multiple reasons through the question: “Considering all the times that you were poorly or unfairly treated because of who you are, with what frequency do you believe that each one of the following factors was a reason others have treated you this way?”. A list of the most common attributions is presented (e.g., age, body size, gender identity or expression, income or economic situation, disability, religion, place of origin, and race or ethnicity) and their frequency is assessed with the response options always, never, or I am not sure.

In a preliminary psychometric analysis, based on respondents from Canada and the United States, the InDI showed good internal and external validity findings with data collected in an online survey ^7^. Even though this publication has not proposed a configural structure for all three measures, the test-retest reliability of each one, evaluated with the intraclass correlation coefficient, was 0.7 (95% confidence interval - 95%CI: 0.6-0.8). For the InDI-A, a one-factor model had good fit to the data, suggesting acceptable to ideal performance of this configural structure; the factor loadings ranged between 0.7 and 0.8. Additionally, the internal consistency of the measure was 0.9, according to Cronbach’s alpha, and the correlation among the items ranged between 0.7 and 0.8. The InDI-D and the InDI-M were not subjected to factorial analysis, but there was strong evidence of construct validity for the three scales, as multiply marginalized participants showed higher frequencies of the three types of discrimination.

The InDI was developed taking into consideration the need to measure experiences with different forms of discrimination and their intersections. This particular characteristic overcomes limitations of widely used discrimination scales that focus on or emphasize experiences with race-based discrimination; this is the case, for example, of the *Everyday Discrimination Scale* ^19^. Considering the good psychometric properties of the English version of the InDI and the recent effort to translate the items into Brazilian Portuguese ^20^, the present study aims to assess configural and metric properties of the InDI in Brazil, following recommendations of the literature on the topic ^21^ ^,^ ^22^.

## Methods

### Data collection, sample, and ethical aspects

The data used in the present study come from a broader investigation, linked to a Doctoral Thesis ^23^ in the field of Psychology, which assessed the mental health impacts from discrimination among Brazilian cisgender women. Selection of participants followed a convenience sampling scheme through social media platforms and email lists. Data collection was carried out online, using Qualtrics software (https://www.qualtrics.com/). Following the advertisement of the study, potentially interested participants would access a link, which would direct them to the research platform where the questionnaire was stored.

In this study, 1,105 women residing in Brazil were approached. Of this initial sample, 1,001 respondents between the ages 18 and 75 (mean = 29.9; standard deviation = 10.1) had valid responses (i.e., at least 45% of the questions properly filled out) and were included in the analysis, corresponding to response rate of 90.5%. Data collection took place from April to July 2021 and was voluntary; all participants signed an informed consent form prior to answering the questionnaire. The study’s protocol was approved by the Research Ethics Committee of the Rio Grande do Sul Pontifical Catholic University, on December 11th, 2020 (protocol number: 4.458.136; CAAE: 40787620.3.0000.5336).

### Measures

Together with the Brazilian Portuguese version of the InDI, information on socioeconomic and demographic characteristics was collected from participants, including place of residence (Brazilian macroregions: Central-West, Northeast, North, Southeast, and South), age, education, sexual orientation, and skin color/race. Age was collected as a continuous variable, and was subsequently categorized as 18-28, 29-39, 40-50 and 51+ years of age. Education was categorized as elementary, secondary, higher education, and graduate degree. Participants also responded if they were heterosexual, bisexual, lesbian, or of another sexual orientation, and were subsequently grouped into two broad categories: sexual minority and non-sexual minority (heterosexual). For skin color/race, the response options were white, black, brown, Indigenous and individuals of Asian descent.

### Data analysis

The sample was randomly divided in two parts of equal size, following the split-half ^24^ procedure. In the first half, the three measures were assessed with exploratory factor analysis (EFA) and exploratory structural equation modelling (ESEM). These models were estimated to determine the number of underlying factors for each measure, the magnitude of the factor loadings, and the residual correlations between specific pairs of items ^24^ ^,^ ^25^. Factor loadings larger than 0.4 were considered acceptable ^21^. Factors were retained with oblique rotation, whenever they presented eigenvalues equal to or greater than 1.0. The most tenable configural and metric structures were then subjected to confirmatory factor analysis (CFA) among the second half of the sample. For each retained factor, average variance extracted (AVE) and the composite reliability (CR) were estimated ^26^.

Modification indices (MIs) and expected parameter change estimates were also calculated to identify better-fitting models with ESEM and CFA, provided that such models had strong theoretical support. The following indicators of overall fit were also estimated: the chi-squared test (lower values indicated better-fitting models), root mean square error of approximation (RMSEA; acceptable values are those below 0.06; the upper bound of the 90% confidence interval - 90%CI - should also be below 0.08), comparative fit index and Tucker-Lewis index (CFI and TLI, respectively; values suggestive of good-fitting models are above 0.95), and the standardized root mean square residual (SRMR; values below 1.0 indicate a good fit to the data) ^24^. Separate analyses were conducted for each measure (i.e., InDI-A, InDI-D, and InDI-M), given that they address distinct processes and research questions. The use of the three measures in the same investigation can increase respondent burden, such that this practice should be avoided ^7^. The cleaning, coding, organization of data, and description of the sample were done with Stata, version 16.1 (https://www.stata.com). EFA, ESEM, and CFA were executed with Mplus, version 8.4 (https://www.statmodel.com/).

## Results

### Characterization of the sample

As can be seen in Table 1, of the 1,001 women who participated in the survey, 67.1% identified as white, 54.7% were between 18-28 years, 43.9% had completed secondary education, and 68.4% did not belong to any sexual minority. The subsamples presented a similar sociodemographic profile. There were responses from 25 out of the 27 Brazilian states, with the majority of observations coming from either the South or the Southeast regions of the country (67.4% of the sample). In what follows, the results of the psychometric analysis are summarized. In order to provide a concise view of the findings, only results from the CFA models are presented in table form. For a comprehensive documentation of the EFA and the ESEM, we recommend consulting the available files at the repository ^27^.

**Table 1:** Socioeconomic and demographic characteristics of the sample.

Characteristics	Total sample	Subsample 1	Subsample 2
	n (%)	n (%)	n (%)
Area of residence			
Central-West	90 (9.0)	44 (8.8)	46 (9.2)
Northeast	162 (16.1)	92 (18.4)	70 (13.9)
North	75 (7.5)	35 (7.0)	40 (8.0)
Southeast	274 (27.4)	134 (26.9)	140 (27.9)
South	400 (40.0)	194 (38.9)	206 (41.0)
Skin color/Race			
White	672 (67.1)	335 (67.2)	337 (67.1)
Brown	191 (19.1)	92 (18.4)	99 (19.7)
Black	105 (10.5)	54 (10.8)	51 (10.2)
Indigenous	7 (0.7)	2 (0.4)	5 (1.0)
Yellow	26 (2.6)	16 (3.2)	10 (2.0)
Age (years)			
18-28	548 (54.7)	273 (54.7)	275 (54.8)
29-39	282 (28.2)	141 (28.3)	141 (28.1)
40-50	124 (12.4)	58 (11.6)	66 (13.1)
51+	47 (4.7)	27 (5.4)	20 (4.0)
Education			
Elementary	10 (0.9)	3 (0.6)	7 (1.4)
Secondary	440 (44.0)	213 (42.7)	227 (45.2)
Higher education	231 (23.1)	124 (24.8)	107 (21.3)
Graduate degree	320 (31.9)	159 (31.9)	161 (32.1)
Sexual orientation			
Sexual non-minority	685 (68.4)	340 (68.2)	345 (68.7)
Sexual minority	316 (31.6)	159 (31.8)	157 (31.3)

### InDI-A

In the EFA of the InDI-A, solutions with up to four factors were examined. The only model that presented a factor with an eigenvalue above 1.0 was the one-factor solution (i.e, 5.2). The unifactorial model had a reasonable fit, with the chi-squared test showing that this model is different from a saturated solution (p-value < 0.05); RMSEA and TLI were suggestive of poor fit (0.12 and 0.94, respectively), even though satisfactory values for CFI and SRMR were observed (0.96 and 0.06, in this order). Overall, the items had moderate to strong loadings, ranging between 0.620 and 0.848.

The ESEM of the one-factor InDI-A had a good fit to the data (chi-square p-value < 0.001; RMSEA = 0.08; CFI = 0.98; TLI = 0.98; and SRMR = 0.03), but also had MI suggesting that residual correlations between two pairs of items (i.e., i3-i5; i7-i8) should be estimated. The unifactorial models with or without residual correlations that emerged from the ESEM were tested in the second half of the sample with CFA (Table 2). When tested, the unifactorial model with no residual correlations presented good fit indices, with the exception of RMSEA and TLI (Model 1, Table 2). The model with a residual correlation between items i7-i8 presented a slightly better fit compared to that which did not contain any residual correlations (RMSEA = 0.10; CFI = 0.97; TLI = 0.96; SRMR = 0.03); such items had a residual correlation of 0.359 (Model 2, Table 2). As observed in Model 3 (Table 2), the inclusion of another residual correlation - between i3-i5 - was associated with an even better fit to the data (RMSEA = 0.08; CFI = 0.98; TLI = 0.97; SRMR = 0,03). Items i7 and i8 had a residual correlation of 0.351, while items i3 and i5, 0.354. The magnitude of residuals for this factorial solution varied between 0.252 and 0.605. In the final step of the analysis, the AVE for this one-factor model was 0.526, and the CR was 0.907.

**Table 2:** Confirmatory factor models for the *Intersectional Discrimination Index* that measures anticipated discrimination (InDI-A).

Items	Model 1		Model 2		Model 3	
	Loadings	Residuals	Loadings	Residuals	Loadings	Residuals
i1: *Talvez algum profissional de saúde (por exemplo, um médico ou enfermeiro) possa me tratar mal.* [Health care provider might treat me poorly.]	0.813	0.339	0.817	0.332	0.824	0.321
i2: *Talvez eu tenha dificuldades para conseguir ou manter um emprego.* [Might have trouble finding or keeping a job.]	0.848	0.281	0.854	0.272	0.865	0.252
i3: *Posso ter problemas para conseguir um apartamento ou casa.* [Might have trouble getting an apartment or house.]	0.831	0.309	0.835	0.303	0.798	0.364
i4: *Eu me preocupo em ser tratado(a) de forma injusta por professores, supervisores ou chefes.* [Worry about being treated unfairly by a teacher, supervisor, or employer.]	0.665	0.558	0.670	0.551	0.676	0.543
i5: *É possível que me seja negada uma conta bancária, empréstimo ou financiamento por ser quem eu sou.* [May be denied a bank account, loan, or mortgage.]	0.745	0.445	0.750	0.438	0.701	0.509
i6: *Eu me preocupo em ser mal tratado(a), ou parado(a) pela polícia ou por seguranças.* [Worry about being harassed or stopped by police or security.]	0.676	0.543	0.681	0.537	0.687	0.528
i7: *As pessoas podem tentar me atacar fisicamente.* [People might try to attack me physically.]	0.674	0.546	0.628	0.606	0.633	0.599
i8: *Eu já espero ser apontado(a), xingado(a), tratado(a) mal ou assediado(a) quando estou em público.* [Expect to be pointed at, called names, or harassed.]	0.672	0.549	0.626	0.608	0.633	0.600
i9: *Tenho medo de ter dificuldade em fazer amigos ou ter um relacionamento íntimo por ser quem eu sou.* [Will have a hard time finding friendship or romance.]	0.620	0.616	0.623	0.611	0.629	0.605
Residual correlations						
i3-i5	-		-		0.354	
i7-i8	-		0.359		0.351	
Fit indexes						
RMSEA	0.122		0.102		0.080	
CFI	0.958		0.972		0.980	
TLI	0.944		0.961		0.971	
SRMR	0.040		0.033		0.030	

CFI: comparative fit index; RMSEA: root mean square error of aproximation; SRMS: standardized root mean square residual; TLI: Tucker-Lewis index.

Note: Model 1 - confirmatory factor analysis (CFA) of the unifactorial model, without residual correlations between pairs of items; Model 2 - CFA of the unifactorial model with residual correlation between items i7-i8; Model 3 - CFA of the unifactorial model with residual correlations between items i3-i5 and i7-i8.

### InDI-D

Solutions with up to three factors were examined for the InDI-D. The only solution that presented an eigenvalue above 1.0 was the unifactorial model. While the items for the one-factor model presented moderate to strong loadings, ranging from 0.693 to 0.795, good fit indices were not observed (chi-square p-value < 0.05; RMSEA = 0.11; CFI = 0.95; TLI = 0.93; SRMR = 0.07).

ESEM suggested that residual correlations between two pairs of items (i.e., i11-i13 and i17-i18) should be estimated, though the model had a reasonable fit to the data (chi-square p-value < 0.001; RMSEA = 0.11; CFI = 0.95; TLI = 0.93; and SRMR = 0.05). Unifactorial models with and without residual correlations were then tested in the second half of the sample, using CFA (Models 1, 2, and 3, Table 3). When tested, Model 1 without residual correlations, had an acceptable fit, except for the TLI, which was 0.934; furthermore, the factor loadings of this solution were moderate to strong. MI revealed expressive residual correlations between the pairs of items i13-i11 and i17-i18. Model 2, which included only the residual correlation between items i11-i13, had a slightly improved fit (RMSEA = 0.09; CFI = 0.97; TLI = 0.96; SRMR = 0.04) compared to the previous model. Once adding the residual correlation between items i17-i18, Model 3 (Table 3) presented an even better fit (RMSEA = 0.06; CFI = 0.98; TLI = 0.98; SRMR = 0.03). The factor loadings for this solution were moderate to strong (ranging between 0.478 and 0.764), and the residual correlations between items i11-i13 (r = 0.442) and i17-i18 (r = 0.366) were of considerable magnitude. The latter model presented the best fit to the data and theoritical support, given the apparent redundancy between i17-i18; AVE and CR were 0.507 and 0.901, respectively. In terms of residuals, these varied between 0.417 and 0.771.

**Table 3:** Confirmatory factor models for the *Intersectional Discrimination Index* that measures day-to-day discrimination (InDI-D).

Items	Model 1		Model 2		Model 3	
	Loadings	Residuals	Loadings	Residuals	Loadings	Residuals
i10: *Ouviu, viu, ou leu outras pessoas fazendo piadas ou rindo de você (ou de pessoas como você).* [Saw others joking or laughing about you.]	0.744	0.446	0.749	0.439	0.756	0.428
i11: *Foi tratado(a) como se fosse uma pessoa agressiva, inútil ou rude.* [Treated as if you are unfriendly, unhelpful, or rude.]	0.721	0.480	0.688	0.527	0.695	0.517
i12: *Foi xingado(a) ou ouviu/viu sua identidade ser usada para ofender alguém.* [Called names or heard/saw your identity used as an insult.]	0.731	0.465	0.735	0.460	0.743	0.448
i13: *Foi tratado(a) como se os outros sentissem medo de você.* [Treated as if others are afraid of you.]	0.544	0.704	0.471	0.778	0.478	0.771
i14: *Foi encarado(a) ou apontado(a) em público.* [Stared or pointed at in public.]	0.751	0.436	0.755	0.430	0.764	0.417
i15: *Ouviu que deveria pensar, agir ou se parecer mais com os outros.* [Should think, act, or look more like others.]	0.733	0.463	0.737	0.456	0.746	0.443
i16: *Ouviu que você ou pessoas como você não pertencem ou não se encaixam em um grupo ou lugar.* [Heard you or people like you don’t belong.]	0.722	0.478	0.726	0.473	0.734	0.462
i17: *Perguntas inapropriadas, ofensivas ou excessivamente pessoais foram feitas.* [Asked inappropriate, offensive, or overly personal questions.]	0.795	0.368	0.800	0.361	0.756	0.429
i18: *Foi tratado(a) como se você fosse menos inteligente ou capaz do que os outros.* [Treated as if you are less smart or capable than others.]	0.693	0.520	0.697	0.514	0.638	0.593
Residual correlations						
i11-i13	-		0.449		0.442	
i17-i18	-		-		0.366	
Fit indexes						
RMSEA	0.114		0.090		0.064	
CFI	0.951		0.970		0.983	
TLI	0.934		0.958		0.976	
SRMR	0.049		0.037		0.031	

CFI: comparative fit index; RMSEA: root mean square error of aproximation; SRMS: standardized root mean square residual; TLI: Tucker-Lewis index.

Note: Model 1 - confirmatory factor analysis (CFA) of the unifactorial model, without residual correlations between items; Model 2 - CFA for the unifactorial model with residual correlation between items i11-i13; Model 3 - CFA of the unifactorial model with residual correlations between items i11-i13 and i17-i18.

### InDI-M

Solutions with up to four factors were examined for the InDI-M. However, factors with eigenvalues above 1.0 were observed only for solutions with one, two, or three factors. The model with only one factor showed good fit (chi-square p-value < 0.001; RMSEA = 0.05; CFI = 0.95; TLI = 0.95; and SRMR = 0.09). Overall, the loadings of this model were moderate, reaching a maximum of 0.774. In particular, item i27 (*Você foi mal tratado(a) de forma repetida no trabalho ou na escola, onde você mora, ou ao utilizar algum tipo de serviço?* [Harassed at work or school, where you live, or when accessing services?]) presented a relatively low loading (i.e., 0.452). The models with two or three factors also showed good fit indices, with the majority of items loading onto only one factor, except for items i28 and i30. Based on these findings, only the unifactorial model was subjected to ESEM and CFA.

The ESEM suggested a residual correlation between items i28 and i30, and had an acceptable fit to the data (chi-square p-value < 0.001; RMSEA = 0.05; CFI = 0.95; TLI = 0.95; and SRMR = 0.08). The two unifactorial models with or without residual correlations that emerged from the ESEM were examined with CFA in the second half of the sample (Table 4). Model 1, without residual correlations, presented a good fit to the data (RMSEA = 0.05; CFI = 0.95; TLI = 0.95; SRMR = 0.08), and moderate to strong factor loadings (between 0.452 and 0.774); MIs suggested that a residual correlation between items i28 and i30 should be estimated. Model 2, which included the residual correlation between items i28 and i30, had an even better fit to the data (RMSEA = 0.03, including the upper limit of the 90% confidence interval, which was below 0.08; CFI = 0.98; TLI = 0.97; SRMR = 0.07), and the factor loadings were moderate to strong (from 0.467 to 0.756); there was a moderate residual correlation between i28-i30 (r = 0.456). The AVE was 0.400, on the other hand, and the CR, 0.876, with relatively high residuals, between 0.429 and 0.782.

**Table 4:** Confirmatory factor models for the *Intersectional Discrimination Index* that measures major discrimination (InDI-M).

Items	Model 1		Model 2	
	Loadings	Residuals	Loadings	Residuals
i19: *Algum profissional de saúde já recusou atendimento para você?* [Health care provider ever refused you care?]	0.665	0.558	0.675	0.545
i20: *Você já foi demitido(a) ou dispensado(a) de um emprego, ou foi recusado(a) para um emprego para o qual foi entrevistado(a)?* [Fired or dismissed from a job, or been turned down for a job?]	0.539	0.709	0.550	0.698
i21: *Você já foi despejado(a) ou teve moradia negada?* [Been evicted or denied housing?]	0.743	0.447	0.756	0.429
i22: *Você já foi injustamente parado(a) e questionado(a), revistado(a) ou preso(a) pela polícia ou segurança?* [Unreasonably stopped and questioned, searched, or arrested by police?]	0.554	0.693	0.567	0.678
i23: *Você já foi injustamente expulso(a) ou suspenso(a) da escola?* [Unreasonably expelled or suspended from school?]	0.565	0.681	0.574	0.671
i24: *Já ocorreu de você não conseguir abrir uma conta bancária, descontar um cheque ou conseguir um empréstimo?* [Unable to open a bank account, cash a check, or get a loan?]	0.530	0.719	0.541	0.707
i25: *Você já teve que se mudar para outro bairro, município, cidade, estado, região ou país?* [Had to move to another neighborhood, town, city, state, province, or country?]	0.700	0.509	0.710	0.496
i26: *Você já perdeu alguma relação próxima (por exemplo, com um membro da família, amizades, parceiro ou parceira)?* [Lost a close relationship?]	0.640	0.590	0.660	0.565
i27: *Você foi mal tratado(a) de forma repetida no trabalho ou na escola, onde você mora, ou ao utilizar algum tipo de serviço?* [Harassed at work or school, where you live, or when accessing services?]	0.452	0.796	0.467	0.782
i28: *Você já foi ameaçado(a) de ataque físico ou sexual?* [Threatened with a physical or sexual attack?]	0.774	0.401	0.694	0.518
i29: *Você já foi atacado(a) fisicamente (por exemplo, cuspido, objetos foram atirados em você, lhe bateram, deram socos, empurrões, puxões ou surras)?* [Been physically attacked?]	0.685	0.531	0.700	0.509
i30: *Já fizeram você se envolver em atividade sexual, ou foi tocado(a) de uma maneira que você não queria?* [Made to engage in sexual activity, or been touched in a sexual way?]	0.625	0.610	0.515	0.735
i31: *Você já passou por alguma situação na qual alguém pegou, danificou ou vandalizou algo de sua propriedade?* [Had someone take, damage, or vandalize your property?]	0.686	0.529	0.702	0.508
Residual correlations				
i28-i30	-		0.456	
Fit indexes				
RMSEA	0.045		0.031	
CFI	0.954		0.978	
TLI	0.450		0.974	
SRMR	0.077		0.071	

CFI: comparative fit index; RMSEA: root mean square error of aproximation; SRMS: standardized root mean square residual; TLI: Tucker-Lewis index.

Nota: Model 1 - confirmatory factor analysis (CFA) of the unifactorial model, without residual correlations between pairs of items; Model 2 - CFA of the unifactorial model with a residual correlation between items i28-i30.

## Discussion

The present study evaluated the configual and metric structures ^21^ ^,^ ^22^ of the InDI in a sample of Brazilian women. In particular, the unifactorial model of the InDI-A with no residual correlations, proposed in the original study ^7^, was not supported by our analysis. Our findings not only suggest that there are potentially redundant pairs of items in the InDI-A, but also that the InDI-D and InDI-M have one-factor structures, some items of which are also potentially redundant. Models with more than one factor were tested; however, these additional dimensions were shown to be spurious and could not be supported by our analysis. Further, the InDI-M presented an AVE below the recommended threshold of 0.500, suggesting that the underlying factor is responsible for less than 50% of the variability of the items.

In the case of the InDI-A, for example, results indicated that the unifactorial solution with residual correlations between items i3 (*Posso ter problemas para conseguir um apartamento ou casa* [Might have trouble getting an apartment or house]) and i5 (*É possível que me seja negada uma conta bancária, empréstimo ou financiamento por ser quem eu sou* [May be denied a bank account, loan, or mortgage]) , as well as i7 (*As pessoas podem tentar me atacar fisicamente* [People might try to attack me physically]) and i8 (*Eu já espero ser apontado(a), xingado(a), tratado(a) mal ou assediado(a) quando estou em público* [Expect to be pointed at. Called names, or harassed]) presented the best fit. This solution was chosen due to the the direct link between items i3 and i5, which reflect how discrimination perpertrated by financial institutions can hinder housing access to marginalized groups. Items i7 and i8 reflect, on the other hand, ties between verbal violence and physical aggression, with the anticipation of the first often preceeding the second.

The InDI-D, in turn, presented better indices of fit and stronger factor loadings for the one-factor solution which included residual correlations between i11-i13 and i17-i18. Items i11 (*Foi tratado(a) como se fosse uma pessoa agressiva, inútil ou rude* [Treated as if you are unfriendly, unhelpful, or rude]) and i13 (*Foi tratado(a) como se os outros sentissem medo de você* [Treated as if others are affraid of you]) could have an overlapping content, in so far as they address the way a person is perceived in relation to aggressive, intimidating, or threatening behavior. Both items refer to how others perceive the individual’s behavior, particularly in relation to aggression, and the ability to cause fear. Yet the residual correlation was weak between items i17 (*Perguntas inapropriadas, ofensivas ou excessivamente pessoais foram feitas* [Asked inappropriated, offensive, or overly personal questions]) and i18 (*Foi tratado(a) como se você fosse menos inteligente ou capaz do que os outros* [Treated as if you are less smart or capable than others]), which could be due to the fact that women are frequently treated as less intelligent or capable through excessive questioning or offensive comments aimed at invalidating their knowledge ^28^, particularly in male-dominated areas ^29^.

Lastly, our findings indicated that the InDI-M has a better fit to the data when the one-factor model with residual correlations between items i28 (*Você já foi ameaçado(a) de ataque físico ou sexual?* [Threatened with a physical or sexual attack?]) and i30 (*Já fizeram você se envolver em atividade sexual, ou foi tocado(a) de uma maneira que você não queria?* [Made to engage in sexual activity, or been touched in a sexual way?]) is estimated. These items are the only ones in the measure that refer to physical aggression or sexual assault, and this might be the reason for their residual correlation; this finding is justified by the fact that the sample is made up of only women, who are often subjected to these types of aggression ^30^.

In conjunction, our findings suggest that the InDI could be characterized by three unidimentional scales with moderate to strong loadings and five pairs of potentially redundant items. Future evaluations of the InDI should address the redundancies identified in the present study. Pairs of items that clearly show overlapping content, such as i3-i5 and i7-i8 (InDI-A), i11-i13 (InDI-D), and i28-i30 (InDI-M), could be combined or have one item excluded from the respective measure. The residual correlation between items that do not appear to have overlapping content (i.e., i17-18 from InDI-D) could be interpreted as stemming from the experiences often shared by Brazilian women in Brazil - inappropriate or offensive questions are often directed at women as a strategy to raise doubts about their intelligence or capabilities. In any case, these and other potentially redundant pairs of items can be evaluated with cognitive interviews ^31^, which will then shed some light on the apparent redundancies.

Despite our comprehensive assessment of the configural and metric properties of the InDI, the present study has some important limitions that must be considered. The sample was restricted to respondents who identified as cisgender. Even though we adopted strategies to increase diversity within the sample, our respondents were mainly white and heterossexual, residing in southeast and south Brazil, with a high level of schooling; this limitation might have implied lower variablity in the responses to the items, attenuating the magnitude of the factor loadings. Other limitations refer to the online data collection procedure, and the dissemination of the research through social media platforms; both may have affected the responses, be it because of the limited access to internet and social media platforms among highly vulnerable women, because the study advertisement was limited and concentrated in the southern and southeastern regions, or even because of limited availability to respond to the online survey. The online administration of the questionnaire was adopted due to the COVID-19 pandemic, which made in-person data collection impossible across institutions that provide services to vulnerable women - the original sampling frame.

The findings documented here represent a futher step in the process of the cross-cultural adaptation of the InDI to the Brazilian context, drawing from a less diverse sample than that used in the translation of the instrument into Portuguese ^20^. In this paper, only cisgender women, diverse in terms of sexual orientation and race/skin color, were surveyed. We hope that this study contributes to the development of other research endeavors adopting an intersectionality framework in the health field, as well as the reduction of health inequities by promoting the visibility of marginalized groups and their experiences.

In conclusion, our analysis points to unifactorial models for each of the three measures with residual correlations between specific pairs of items. In the InDI-A, the residual correlation was observed between items related to the expectation of financial discrimination and difficulty finding housing, as well as anticipating verbal or physical agression. In the InDI-D, the potentially redundant items addressed the respondent’s perception of being an aggressive or a violent person, and how minority groups are often questioned and perceived to be less able. In the InDI-M, in turn, the residual correlation was observed between items that address physical and sexual assault. Further assessment of redundancy is recommended for the pairs of items with expressive residual correlations. In addition, future studies should examine these and other properties among broader samples, including invariance between groups defined on the basis of gender, social class, age, sexual orientation, race, and their intersections.
