# Supplementary material for: Psychometric evaluation of the Intersectional Discrimination Index for use in Brazil
Source: Cad Saude Publica. 2024 Sep 23;40(9):e00009724. doi: 10.1590/0102-311XEN009724 (PMC11423766; doi:10.1590/0102-311XEN009724)
Supplement: Translation

# Translation: Avaliação psicométrica do *Intersectional Discrimination Index* para uso no Brasil

**DOI:** 10.1590/0102-311XPT009724

**Authors:** Natália Peixoto Pereira, Carolina Saraiva de Macedo Lisboa, João Luiz Bastos

**Affiliations:** 1 Pontifícia Universidade Católica do Rio Grande do Sul, Porto Alegre, Brasil.; 2 Simon Fraser University, Burnaby, Canada.

**Keywords:** Enquadramento Interseccional, Discriminação Percebida, Psicometria, Análise Fatorial

## Abstract

Este estudo transversal avaliou as estruturas configural e métrica do *Intersectional Discrimination Index* (InDI), um instrumento que afere discriminação antecipada (InDI-A), cotidiana (InDI-D) e maior (InDI-M). Dados de uma pesquisa mais ampla, voltada para os impactos da discriminação na saúde mental de mulheres residentes no Brasil, foram utilizados. Aproximadamente mil mulheres, selecionadas por conveniência, responderam ao InDI e a perguntas sobre características sociodemográficas em formulário eletrônico, aplicado em 2021. Enquanto na primeira metade da amostra foram realizadas análises fatoriais exploratórias e executada modelagem por equações estruturais exploratórias, na segunda foi conduzida análise fatorial confirmatória. Em conjunto, os achados sugerem que cada uma das três medidas é unidimensional. No entanto, diferentemente do estudo que originalmente propôs o InDI para uso no Canadá e nos Estados Unidos, observamos a presença de correlações residuais nas três subescalas avaliadas, todas elas sugestivas de redundância de conteúdo entre pares específicos de itens. As três medidas apresentaram cargas fatoriais moderadas a fortes e índices aceitáveis de ajuste. O InDI exibiu indicadores de validade interna razoáveis, potencialmente se tornando um valioso instrumento para a investigação dos efeitos para a saúde da discriminação interseccional no Brasil. Estudos futuros devem avaliar a consistência desses achados, examinar a estrutura escalar do instrumento e analisar sua invariância entre diferentes grupos marginalizados.

## Introdução

A discriminação se refere a um processo em que membros de um grupo socialmente definido são tratados de forma diferente e injusta, simplesmente por fazerem parte do segmento populacional em questão ^1^. Desde os anos 1990, um crescente corpo de pesquisa tem relacionado a discriminação com desfechos negativos e iniquidades em saúde ^2^. A maior parte dessa literatura se concentra nos prejuízos para a saúde da discriminação étnico-racial, embora também haja evidências robustas sobre os efeitos deletérios da discriminação com base em gênero, orientação sexual, deficiência, condição de saúde mental e tamanho corporal ^3^ ^,^ ^4^ ^,^ ^5^ ^,^ ^6^. Além de pesquisas destinadas a avaliar discriminação por diferentes motivações ^4^, é crescente o interesse em investigar circunstâncias em que múltiplas formas de discriminação se combinam e suas consequências para a saúde ^7^.

Compreender o papel da discriminação na produção e manutenção de iniquidades em saúde representa uma área importante de pesquisa, tendo em vista que é um processo passível de redução ou eliminação ^8^. Entretanto, ainda são necessários estudos que analisem as experiências de grupos que estão nas intersecções de raça, gênero, classe, além de outros marcadores de injustiça social ^9^. A interseccionalidade tem sido utilizada para lidar com essas questões, na medida em que se propõe a investigar as relações entre poder, sistemas de opressão, identidades e processos de marginalização ^10^ ^,^ ^11^, com aplicação recente em áreas específicas do conhecimento científico, incluindo a saúde coletiva ^8^ ^,^ ^10^ ^,^ ^12^.

Em particular, a relação entre diferentes sistemas de opressão é pautada por teóricas feministas negras, dos Estados Unidos, há bastante tempo ^13^, antes mesmo de o termo interseccionalidade ter sido cunhado por Kimberlé Crenshaw ^14^. Tal conceito foi elaborado para descrever as experiências de discriminação de mulheres negras estadunidenses no final da década de 1980. Estudiosos da interseccionalidade argumentam que a análise interseccional privilegia as experiências de grupos que enfrentam formas de discriminação cruzadas, que não podem ser isoladas umas das outras ^8^ ^,^ ^10^. Ainda que a quantidade de estudos adotando uma perspectiva interseccional tenha aumentado expressivamente nos últimos anos ^15^ ^,^ ^16^, pesquisadores da Saúde Coletiva devem enfrentar alguns desafios, como a necessidade de: (1) desenvolver instrumentos que permitam aferir a discriminação interseccional; (2) elucidar mecanismos explicativos para as iniquidades em saúde; e (3) investigar grupos caracterizados por outros marcadores de injustiça, além de raça, gênero e orientação sexual.

Pesquisas recentes visaram enfrentar alguns desses desafios por meio da elaboração de instrumentos capazes de aferir experiências com diferentes formas de discriminação. Um desses é o *Intersectional Discrimination Index* (InDI), originalmente proposto no Canadá e nos Estados Unidos por Scheim & Bauer, em 2019 ^7^. O InDI é um instrumento de autorrelato com 31 itens distribuídos em três subescalas que buscam mensurar discriminação antecipada (InDI-A), cotidiana (InDI-D) e maior (InDI-M). Para limitar a atenção ao tratamento discriminatório sem qualquer tipo de atribuição, o InDI questiona sobre experiências com tratamento injusto “por causa de quem você é”. A seguinte definição é fornecida aos respondentes: “Essas perguntas são sobre experiências relacionadas a quem você é. Isso inclui como você se descreve e como os outros podem descrevê-lo. Por exemplo, sua cor de pele, ancestralidade, nacionalidade, religião, gênero, sexualidade, idade, peso, deficiência ou problema de saúde mental e renda”.

O InDI-A tem nove itens avaliados em uma escala de Likert de cinco pontos, variando de 0 (discordo totalmente) a 4 (concordo totalmente). Essa subescala baseia-se em teorias de estresse de minorias, as quais apregoam que a constante exposição à discriminação faz com que os indivíduos fiquem vigilantes, antecipando tais experiências ^7^ ^,^ ^17^. Por sua vez, o InDI-D possui nove itens avaliados em uma escala de frequência de quatro pontos, sendo as opções de resposta “nunca”, “sim, mas não no ano passado”, “sim, uma vez no ano passado” e “sim, muitas vezes no ano passado”. Esses itens foram desenvolvidos com base em medidas que avaliam microagressões (e.g., pequenos insultos e invalidações), capacitismo, gordofobia, racismo, LGBTfobia e discriminação interseccional, entre outros ^7^. Por fim, o InDI-M é composto por 13 itens, os quais são avaliados em uma escala de frequência com as seguintes opções de resposta: “nunca”, “uma vez” e “mais de uma vez”. Tendo em vista que o item nove do InDI-M aborda especificamente assédio repetido, ele apresenta como opções de resposta “não”, “sim, em um lugar” e “sim, em mais de um lugar”. No InDI-M, é igualmente analisado se as experiências com discriminação ocorreram nos últimos 12 meses. Os itens de discriminação maior foram adaptados de outras escalas ^18^ e tratam de situações extremas, tais como negação de serviço, danos à propriedade e violência física.

Na tentativa de documentar formas específicas de discriminação, o InDI também possibilita ao respondente atribuir suas experiências com discriminação a diversas razões por meio da pergunta: “Pensando em todas as vezes em que você foi tratado injustamente ou mal por causa de quem você é, com que frequência você acha que cada um dos fatores a seguir foi um motivo pelo qual os outros o trataram dessa forma?”. Uma lista com as atribuições mais comuns é apresentada (e.g., idade, tamanho corporal, identidade ou expressão de gênero, renda ou situação econômica, deficiência, religião, local de origem, raça, etnia) e suas frequências são avaliadas com as opções de resposta “sempre”, “nunca” ou “não tenho certeza”.

Numa análise psicométrica preliminar, baseada em respondentes do Canadá e dos Estados Unidos, o InDI apresentou bons resultados de validade interna e externa com dados coletados em pesquisa *online* ^7^. Ainda que essa publicação não tenha proposto um modelo estrutural para todas as três medidas do instrumento, a confiabilidade teste-reteste de cada uma delas, avaliada pelo coeficiente de correlação intraclasse, foi de 0,7 (intervalo de 95% de confiança - IC95%: 0,6-0,8). Para o InDI-A, o ajuste do modelo unifatorial foi bom, sugerindo desempenho aceitável a ideal dessa estrutura configural; as cargas fatoriais dos itens variaram de 0,7 a 0,8. Além disso, a consistência interna dessa medida foi de 0,9, segundo o alfa de Cronbach, e as correlações totais entre os itens variaram de 0,7 a 0,8. O InDI-D e o InDI-M não foram submetidos a análises fatoriais, mas foram observadas boas evidências de validade de construto para as três medidas, uma vez que os participantes multiplamente marginalizados revelaram frequências mais altas dos três tipos de discriminação.

O InDI foi desenvolvido levando em consideração a necessidade de captar experiências com diferentes tipos de discriminação e suas intersecções. Essa característica enfrenta críticas recentes sobre o fato de escalas de discriminação amplamente utilizadas terem o foco em experiências de racismo; esse é o caso, por exemplo, do *Everyday Discrimination Scale* ^19^. Tendo em vista as boas propriedades psicométricas da versão em inglês do InDI e o recente esforço de tradução de seus itens para o português do Brasil ^20^, este estudo tem por objetivo dar continuidade ao processo de avaliação psicométrica do instrumento no país, enfatizando suas estruturas configural e métrica, conforme preconizado na literatura sobre o assunto ^21^ ^,^ ^22^.

## Métodos

### Coleta de dados, amostra e aspectos éticos

Os dados utilizados neste artigo provêm de uma pesquisa mais ampla, vinculada a uma tese de doutorado ^23^ no âmbito da Psicologia, a qual visou avaliar os efeitos da discriminação na saúde mental de mulheres cisgênero brasileiras. O recrutamento das participantes ocorreu por meio de amostragem por conveniência, através da divulgação da pesquisa em mídias sociais e listas de e-mail. A coleta de dados foi realizada de forma *online*, na plataforma Qualtrics (https://www.qualtrics.com/). Após a divulgação da pesquisa, as mulheres interessadas em participar acessaram um *link* que as direcionou para a plataforma de pesquisa, na qual o questionário estava armazenado.

No estudo, foram abordadas 1.105 mulheres, residentes no Brasil. Desse total, 1.001 respondentes, com idade entre 18 anos e 75 anos (média = 29,9; desvio padrão = 10,1) apresentaram respostas válidas (i.e., pelo menos 45% das perguntas devidamente preenchidas) e foram incluídas nas análises, correspondendo a uma taxa de resposta de 90,5%. A coleta de dados ocorreu no período de abril a julho de 2021 e foi realizada de forma voluntária, sendo que todas as participantes assinaram um termo de consentimento informado antes do preenchimento do questionário. O protocolo do estudo foi aprovado pelo Comitê de Ética em Pesquisa da Pontifícia Universidade Católica do Rio Grande do Sul em 11 de dezembro de 2020 (protocolo: 4.458.136; CAAE: 40787620.3.0000.5336).

### Medidas

Juntamente com o InDI traduzido para o português do Brasil ^20^, foram administradas perguntas de natureza socioeconômica e demográfica para caracterizar as participantes quanto a macrorregião de moradia (i.e., Centro-oeste, Nordeste, Norte, Sudeste e Sul), idade, escolaridade, orientação sexual e cor/raça. A idade foi coletada como uma variável contínua, sendo posteriormente categorizada em faixas etárias de 18-28, 29-39, 40-50 e 51+ anos. A escolaridade foi, por sua vez, agrupada em Ensino Fundamental, Ensino Médio, Ensino Superior e Pós-graduação. As participantes também responderam se eram heterossexuais, bissexuais, lésbicas ou de outra orientação sexual, tendo sido agrupadas em duas categorias a partir das respostas: minoria sexual e não minoria sexual (heterossexual). Para cor/raça, as opções de resposta incluíram branca, preta, parda, indígena e amarela (i.e., indivíduos de origem asiática).

### Análise dos dados

A amostra foi dividida aleatoriamente em duas partes de semelhante tamanho, seguindo o procedimento *split-half* ^24^. Na primeira metade, as três subescalas foram examinadas com a análise fatorial exploratória (AFE) e modelos exploratórios de equações estruturais (MEEE). Esses modelos foram estimados para determinar o número de fatores subjacentes a cada medida de discriminação, a magnitude das cargas fatoriais e a ocorrência de correlações residuais entre pares específicos de itens ^24^ ^,^ ^25^. Cargas fatoriais acima de 0,4 foram consideradas aceitáveis ^21^. Os fatores foram retidos por meio de rotação oblíqua, sempre que apresentavam autovalores iguais ou superiores a 1,0. As estruturas configural e métrica mais verossímeis foram então submetidas à análise fatorial confirmatória (AFC) na segunda metade da amostra. Para cada fator retido e analisado com AFC, foram estimadas a variância média extraída (VME) e a confiabilidade composta (CC) ^26^.

Índices de modificação (IM) e estimativas de mudanças esperadas nos parâmetros de interesse também foram calculados para identificar modelos alternativos com melhor ajuste na MEEE e AFC, desde que esses tivessem respaldo teórico. Foram igualmente estimados os seguintes indicadores de ajuste global: o teste qui-quadrado do modelo e do modelo basilar (valores menores indicam melhor ajuste), erro quadrático médio de aproximação (RMSEA, acrônimo em inglês; valores aceitáveis são aqueles abaixo de 0,06, além de o limite superior do intervalo de 90% de confiança - IC90% - estar abaixo de 0,08), índice de ajuste comparativo e índice Tucker-Lewis (CFI e TLI, respectivamente, acrônimos em inglês; valores sugestivos de bom ajuste estão acima de 0,95) e a raiz quadrada média residual padronizada (SRMR, acrônimo em inglês; valores abaixo de 1,0 indicam um ajuste adequado) ^24^. Análises separadas foram conduzidas para cada medida de discriminação (ou seja, InDI-A, InDI-D e InDI-M), dado que abordam processos reconhecidamente distintos e respondem a perguntas de pesquisa diversas. A utilização das três medidas de discriminação em conjunto em uma mesma investigação pode gerar um impacto maior nos participantes, sendo, portanto, recomendado evitar tal prática ^7^. A limpeza, a codificação, a organização dos dados e a descrição da amostra foram realizadas no Stata, versão 16.1 (https://www.stata.com). Foram executadas com o Mplus, versão 8.4 (https://www.statmodel.com/), AFE, MEEE e AFC.

## Resultados

### Caracterização da amostra

Conforme pode ser observado na Tabela 1, das 1.001 mulheres que participaram da pesquisa, 67,1% se declararam brancas, 54,7% informaram ter entre 18-28 anos, 43,9% afirmaram ter o Ensino Médio completo e 68,4% não pertenciam a nenhuma minoria sexual. Ambas as subamostras apresentaram perfil sociodemográfico semelhante. Das 27 Unidades da Federação, obtiveram-se respondentes de 25, havendo concentração dessas nas regiões Sul e Sudeste do país (67,4% da amostra). A seguir são detalhados os resultados das análises psicométricas. Com o propósito de oferecer uma visão concisa dos achados, estão apresentadas em tabelas apenas as estimativas derivadas dos modelos de AFC. Para uma documentação mais abrangente da AFE e da MEEE, recomendamos consultar os documentos disponíveis em repositório ^27^.

**Tabela 1:** Características socioeconômicas e demográficas da amostra.

Características	Amostra total	Subamostra 1	Subamostra 2
n (%)	n (%)	n (%)
Região de moradia			
Centro-oeste	90 (9,0)	44 (8,8)	46 (9,2)
Nordeste	162 (16,1)	92 (18,4)	70 (13,9)
Norte	75 (7,5)	35 (7,0)	40 (8,0)
Sudeste	274 (27,4)	134 (26,9)	140 (27,9)
Sul	400 (40,0)	194 (38,9)	206 (41,0)
Cor/Raça			
Branca	672 (67,1)	335 (67,2)	337 (67,1)
Parda	191 (19,1)	92 (18,4)	99 (19,7)
Preta	105 (10,5)	54 (10,8)	51 (10,2)
Indígena	7 (0,7)	2 (0,4)	5 (1,0)
Amarela	26 (2,6)	16 (3,2)	10 (2,0)
Faixa etária (anos)			
18-28	548 (54,7)	273 (54,7)	275 (54,8)
29-39	282 (28,2)	141 (28,3)	141 (28,1)
40-50	124 (12,4)	58 (11,6)	66 (13,1)
51+	47 (4,7)	27 (5,4)	20 (4,0)
Escolaridade			
Ensino Fundamental	10 (0,9)	3 (0,6)	7 (1,4)
Ensino Médio	440 (44,0)	213 (42,7)	227 (45,2)
Ensino Superior	231 (23,1)	124 (24,8)	107 (21,3)
Pós-graduação	320 (31,9)	159 (31,9)	161 (32,1)
Orientação sexual			
Não minoria sexual	685 (68,4)	340 (68,2)	345 (68,7)
Minoria sexual	316 (31,6)	159 (31,8)	157 (31,3)

### InDI-A

Na AFE do InDI-A, foram examinadas soluções com até quatro fatores. No entanto, a única solução que apresentou fator com autovalor acima de 1,0 foi o modelo unidimensional (i.e., 5,2). O modelo unifatorial teve ajuste razoável, com qui-quadrado indicando que esse é diferente da solução saturada (p < 0,05); RMSEA e TLI foram sugestivos de ajuste inadequado (0,12 e 0,94, respectivamente), ainda que valores satisfatórios de CFI e SRMR tenham sido observados (0,96 e 0,06, nessa ordem). De maneira geral, os itens tiveram cargas moderadas a fortes, variando entre 0,620 e 0,848.

A MEEE do InDI-A com um único fator apresentou IM sugerindo correlações residuais entre dois pares de itens (i.e., i3-i5; i7-i8) e ajuste razoável para essa solução (qui-quadrado p < 0,001; RMSEA = 0,08; CFI = 0,98; TLI = 0,98; e SRMR = 0,03). Os modelos de um fator com e sem as correlações residuais que emergiram da MEEE foram testados na segunda metade da amostra com AFC (Tabela 2). Ao ser testado, o modelo unifatorial sem correlação residual apresentou bons índices de ajuste, com exceção do RMSEA e do TLI (Modelo 1, Tabela 2). O modelo com a correlação residual entre os itens i7-i8 apresentou ligeira melhora no ajuste em relação àquele que não continha qualquer correlação residual (RMSEA = 0,10; CFI = 0,97; TLI = 0,96; SRMR = 0,03); tais itens tiveram correlação residual de 0,359 (Modelo 2, Tabela 2). Conforme observado no Modelo 3 (Tabela 2), a inclusão de mais uma correlação residual - entre i3-i5 - esteve relacionada com ainda melhor ajuste (RMSEA = 0,08; CFI = 0,98; TLI = 0,97; SRMR = 0,03). O par i7-i8 teve correlação residual de 0,351, enquanto o i3-i5, de 0,354. Por sua vez, a magnitude dos resíduos dessa solução fatorial variou entre 0,252 e 0,605. Em etapa final da análise, observou-se que a VME para esse fator único foi de 0,526, ao passo que a CC atingiu o valor de 0,907.

**Tabela 2:** Modelos de análise fatorial confirmatória para o *Intersectional Discrimination Index* que afere discriminação antecipada (InDI-A).

Itens	Modelo 1		Modelo 2		Modelo 3	
	Cargas	Resíduos	Cargas	Resíduos	Cargas	Resíduos
i1: Talvez algum profissional de saúde (por exemplo, um médico ou enfermeiro) possa me tratar mal.	0,813	0,339	0,817	0,332	0,824	0,321
i2: Talvez eu tenha dificuldades para conseguir ou manter um emprego.	0,848	0,281	0,854	0,272	0,865	0,252
i3: Posso ter problemas para conseguir um apartamento ou casa.	0,831	0,309	0,835	0,303	0,798	0,364
i4: Eu me preocupo em ser tratado(a) de forma injusta por professores, supervisores ou chefes.	0,665	0,558	0,670	0,551	0,676	0,543
i5: É possível que me seja negada uma conta bancária, empréstimo ou financiamento por ser quem eu sou.	0,745	0,445	0,750	0,438	0,701	0,509
i6: Eu me preocupo em ser mal tratado(a), ou parado(a) pela polícia ou por seguranças.	0,676	0,543	0,681	0,537	0,687	0,528
i7: As pessoas podem tentar me atacar fisicamente.	0,674	0,546	0,628	0,606	0,633	0,599
i8: Eu já espero ser apontado(a), xingado(a), tratado(a) mal ou assediado(a) quando estou em público.	0,672	0,549	0,626	0,608	0,633	0,600
i9: Tenho medo de ter dificuldade em fazer amigos ou ter um relacionamento íntimo por ser quem eu sou.	0,620	0,616	0,623	0,611	0,629	0,605
Correlações residuais						
i3-i5	-		-		0,354	
i7-i8	-		0,359		0,351	
Índices de ajuste						
RMSEA	0,122		0,102		0,080	
CFI	0,958		0,972		0,980	
TLI	0,944		0,961		0,971	
SRMR	0,040		0,033		0,030	

CFI: índice de ajuste comparativo; RMSEA: erro quadrático médio de aproximação; SRMS: raiz quadrada média residual padronizada; TLI: índice Tucker-Lewis.

Nota: Modelo 1 - análise fatorial confirmatória (AFC) de modelo unifatorial, sem correlações residuais entre pares de itens; Modelo 2 - AFC de modelo unifatorial com correlação residual entre itens i7-i8; Modelo 3 - AFC de modelo unifatorial com correlação residual entre itens i3-i5 e i7-i8.

### InDI-D

Foram examinadas soluções com até três fatores para o InDI-D. A única solução que apresentou autovalor acima de 1,0 foi o modelo unifatorial. Embora os itens do modelo de um fator tenham apresentado cargas fatoriais moderadas a fortes, variando de 0,693 a 0,795, não foram observados bons índices de ajuste (qui-quadrado p < 0,05; RMSEA = 0,11; CFI = 0,95; TLI = 0,93; SRMR = 0,07).

No contexto de MEEE, a análise do InDI-D sugeriu correlações residuais entre dois pares de itens (i.e., i11-i13 e i17-i18) e revelou um ajuste razoável aos dados (qui-quadrado p < 0,001; RMSEA = 0,11; CFI = 0,95; TLI = 0,93; e SRMR = 0,05). Modelos unifatoriais com e sem correlações residuais foram então testados na segunda metade da amostra, por meio de AFC (Modelos 1, 2 e 3, Tabela 3). Quando testado, o Modelo 1, sem correlações residuais, teve ajuste aceitável, exceto pelo TLI de 0,934; além disso, as cargas fatoriais dessa solução foram moderadas a fortes. Índices de modificação sugeriram correlações residuais expressivas entre os pares de itens i13-i11 e i17-i18. O Modelo 2, que incluiu apenas a correlação residual entre os itens i11-i13, teve ajuste ligeiramente melhor (RMSEA = 0,09; CFI = 0,97; TLI = 0,96; SRMR = 0,04) do que o modelo anterior. Ao ser adicionada a correlação residual entre os itens i17-i18, o Modelo 3 (Tabela 3) apresentou ainda melhor ajuste (RMSEA = 0,06; CFI = 0,98; TLI = 0,98; SRMR = 0,03). As cargas fatoriais dessa solução foram moderadas a fortes (com amplitude entre 0,478 e 0,764), e as correlações residuais entre os itens i11-i13 (r = 0,442) e i17-i18 (r = 0,366), expressivas. Esse último modelo foi o que apresentou o melhor ajuste e respaldo teórico, especialmente em função da aparente redundância de conteúdo entre i17-i18 e das estimativas de VME e CC, as quais foram 0,507 e 0,901, respectivamente. Em termos de resíduo, esses variaram entre 0,417 e 0,771.

**Tabela 3:** Modelos de análise fatorial confirmatória para o *Intersectional Discrimination Index* que afere discriminação cotidiana (InDI-D).

Itens	Modelo 1		Modelo 2		Modelo 3	
	Cargas	Resíduos	Cargas	Resíduos	Cargas	Resíduos
i10: Ouviu, viu, ou leu outras pessoas fazendo piadas ou rindo de você (ou de pessoas como você).	0,744	0,446	0,749	0,439	0,756	0,428
i11: Foi tratado(a) como se fosse uma pessoa agressiva, inútil ou rude.	0,721	0,480	0,688	0,527	0,695	0,517
i12: Foi xingado(a) ou ouviu/viu sua identidade ser usada para ofender alguém.	0,731	0,465	0,735	0,460	0,743	0,448
i13: Foi tratado(a) como se os outros sentissem medo de você.	0,544	0,704	0,471	0,778	0,478	0,771
i14: Foi encarado(a) ou apontado(a) em público.	0,751	0,436	0,755	0,430	0,764	0,417
i15: Ouviu que deveria pensar, agir ou se parecer mais com os outros.	0,733	0,463	0,737	0,456	0,746	0,443
i16: Ouviu que você ou pessoas como você não pertencem ou não se encaixam em um grupo ou lugar.	0,722	0,478	0,726	0,473	0,734	0,462
i17: Perguntas inapropriadas, ofensivas ou excessivamente pessoais foram feitas.	0,795	0,368	0,800	0,361	0,756	0,429
i18: Foi tratado(a) como se você fosse menos inteligente ou capaz do que os outros.	0,693	0,520	0,697	0,514	0,638	0,593
Correlações residuais						
i11-i13	-		0,449		0,442	
i17-i18	-		-		0,366	
Índices de ajuste						
RMSEA	0,114		0,090		0,064	
CFI	0,951		0,970		0,983	
TLI	0,934		0,958		0,976	
SRMR	0,049		0,037		0,031	

CFI: índice de ajuste comparativo; RMSEA: erro quadrático médio de aproximação; SRMS: raiz quadrada média residual padronizada; TLI: índice Tucker-Lewis.

Nota: Modelo 1 - análise fatorial confirmatória (AFC) de modelo unifatorial, sem correlações residuais entre pares de itens; Modelo 2 - AFC de modelo unifatorial com correlação residual entre itens i11-i13; Modelo 3 - AFC de modelo unifatorial com correlação residual entre itens i11-i13 e i17-i18.

### InDI-M

Foram examinadas soluções com até quatro fatores para o InDI-M. No entanto, as que demonstraram autovalores acima de 1,0 foram apenas aquelas com um, dois e três fatores. O modelo com um único fator mostrou-se bem ajustado (qui-quadrado p < 0,001; RMSEA = 0,05; CFI = 0,95; TLI = 0,95; e SRMR = 0,09). Em geral, as cargas desse modelo foram moderadas, atingindo um máximo de 0,774. Em particular, o item i27 (Você foi maltratado(a) de forma repetida no trabalho ou na escola, onde você mora, ou ao utilizar algum tipo de serviço?), apresentou carga relativamente baixa (i.e., 0,452). Os modelos com dois e três fatores também mostraram bons índices de ajuste, com a maioria dos itens carregando em um único fator, exceto i28 e i30. Com base nesses resultados, apenas o modelo unifatorial foi submetido à MEEE e AFC.

A MEEE indicou correlação residual entre o par i28-i30 e ajuste aceitável (qui-quadrado p < 0,001; RMSEA = 0,05; CFI = 0,95; TLI = 0,95; e SRMR = 0,08). Os dois modelos unifatoriais com e sem as correlações residuais que emergiram da MEEE foram testados por meio de CFA na segunda metade da amostra (Tabela 4). O Modelo 1, sem correlações residuais, apresentou bom ajuste aos dados (RMSEA = 0,05; CFI = 0,95; TLI = 0,95; SRMR = 0,08) e cargas fatoriais moderadas a fortes (entre 0,452 e 0,774), com o IM sugerindo correlação entre os itens i28-i30. O Modelo 2, que incluiu a correlação entre os itens i28 e i30, teve ainda melhor ajuste (RMSEA = 0,03, incluindo o limite superior do IC90% abaixo de 0,08; CFI = 0,98; TLI = 0,97; SRMR = 0,07) e cargas fatoriais moderadas a fortes (de 0,467 a 0,756); a correlação entre i28-i30 foi moderada (r = 0,456). Por outro lado, a VME alcançou apenas 0,400 e a CC, 0,876, com valores de resíduos relativamente elevados, entre 0,429 e 0,782.

**Tabela 4:** Modelos de análise fatorial confirmatória do *Intersectional Discrimination Index* que afere discriminação maior (InDI-M).

Itens	Modelo 1		Modelo 2	
	Cargas	Resíduos	Cargas	Resíduos
i19: Algum profissional de saúde já recusou atendimento para você?	0,665	0,558	0,675	0,545
i20: Você já foi demitido(a) ou dispensado(a) de um emprego, ou foi recusado(a) para um emprego para o qual foi entrevistado(a)?	0,539	0,709	0,550	0,698
i21: Você já foi despejado(a) ou teve moradia negada?	0,743	0,447	0,756	0,429
i22: Você já foi injustamente parado(a) e questionado(a), revistado(a) ou preso(a) pela polícia ou segurança?	0,554	0,693	0,567	0,678
i23: Você já foi injustamente expulso(a) ou suspenso(a) da escola?	0,565	0,681	0,574	0,671
i24: Já ocorreu de você não conseguir abrir uma conta bancária, descontar um cheque ou conseguir um empréstimo?	0,530	0,719	0,541	0,707
i25: Você já teve que se mudar para outro bairro, município, cidade, estado, região ou país?	0,700	0,509	0,710	0,496
i26: Você já perdeu alguma relação próxima (por exemplo, com um membro da família, amizades, parceiro ou parceira)?	0,640	0,590	0,660	0,565
i27: Você foi mal tratado(a) de forma repetida no trabalho ou na escola, onde você mora, ou ao utilizar algum tipo de serviço?	0,452	0,796	0,467	0,782
i28: Você já foi ameaçado(a) de ataque físico ou sexual?	0,774	0,401	0,694	0,518
i29: Você já foi atacado(a) fisicamente (por exemplo, cuspido, objetos foram atirados em você, lhe bateram, deram socos, empurrões, puxões ou surras)?	0,685	0,531	0,700	0,509
i30: Já fizeram você se envolver em atividade sexual, ou foi tocado(a) de uma maneira que você não queria?	0,625	0,610	0,515	0,735
i31: Você já passou por alguma situação na qual alguém pegou, danificou ou vandalizou algo de sua propriedade?	0,686	0,529	0,702	0,508
Correlações residuais				
i28-i30	-		0,456	
Índices de ajuste				
RMSEA	0,045		0,031	
CFI	0,954		0,978	
TLI	0,450		0,974	
SRMR	0,077		0,071	

CFI: índice de ajuste comparativo; RMSEA: erro quadrático médio de aproximação; SRMS: raiz quadrada média residual padronizada; TLI: índice Tucker-Lewis.

Nota: Modelo 1 - Análise fatorial confirmatória (AFC) de modelo unifatorial, sem correlações residuais entre pares de itens; Modelo 2 - AFC de modelo unifatorial com correlação residual entre itens i28-i30.

## Discussão

Este estudo avaliou as estruturas configural e métrica ^21^ ^,^ ^22^ do InDI em uma amostra de mulheres brasileiras, selecionadas por conveniência. Em particular, o modelo unifatorial e sem correlações residuais do InDI-A, proposto no estudo original ^7^, não foi confirmado na análise. Nossos achados não apenas sugeriram que há itens potencialmente redundantes no InDI-A, mas também que o InDI-D e o InDI-M apresentam estruturas unifatoriais, cujos itens são afetados por problemas semelhantes. Modelos com mais de um fator foram testados, embora essas dimensões adicionais tenham se mostrado espúrias e não puderam ser sustentadas ou recomendadas após apreciação. Ainda assim, o InDI-M apresentou AVE abaixo do patamar recomendado de 0,500, sugerindo que o único fator subjacente é responsável por menos de 50% da variabilidade de seus itens.

No caso do InDI-A, por exemplo, os resultados deste estudo indicaram que a solução unifatorial com correlações residuais entre os itens i3 (Posso ter problemas para conseguir um apartamento ou casa) e i5 (É possível que me seja negada uma conta bancária, empréstimo ou financiamento por ser quem eu sou), bem como entre i7 (As pessoas podem tentar me atacar fisicamente) e i8 (Eu já espero ser apontado(a), xingado(a), tratado(a) mal ou assediado(a) quando estou em público) apresentou o melhor ajuste. Essa solução foi escolhida devido à relação direta entre os itens i3-i5, que refletem como a discriminação perpetrada por instituições financeiras pode dificultar o acesso à moradia de grupos marginalizados. Já os itens i7-i8 refletem vínculos entre violência verbal e agressão física, com a antecipação da primeira frequentemente precedendo a segunda.

O InDI-D, por sua vez, apresentou melhores índices de ajuste e cargas fatoriais na solução de um fator com correlações residuais entre i11-13 e 17-18. Os itens i11 (Foi tratado(a) como se fosse uma pessoa agressiva, inútil ou rude) e i13 (Foi tratado(a) como se os outros sentissem medo de você) podem ter sobreposição de conteúdo, na medida em que abordam a maneira como uma pessoa é percebida em relação a comportamentos agressivos, intimidantes ou ameaçadores. Ambos os itens se referem à forma como os outros percebem o comportamento do indivíduo, particularmente em relação à agressividade ou à capacidade de gerar medo. Embora tenha sido fraca, a correlação residual entre os itens i17 (Perguntas inapropriadas, ofensivas ou excessivamente pessoais foram feitas) e i18 (Foi tratado(a) como se você fosse menos inteligente ou capaz do que os outros) pode se dever ao fato de que mulheres são frequentemente tratadas como menos inteligentes ou capazes por meio de perguntas excessivas e ou de comentários ofensivos que visam questionar e inferiorizar seu conhecimento ^28^, principalmente em áreas dominadas por homens ^29^.

Por fim, os resultados das análises indicaram que o InDI-M tem melhor ajuste no modelo de um fator com as correlações residuais entre os itens i28 (Você já foi ameaçado(a) de ataque físico ou sexual?) e i30 (Já fizeram você se envolver em atividade sexual, ou foi tocado(a) de uma maneira que você não queria?). Esses itens são os únicos da subescala que se referem à agressão física ou ao assédio sexual, e sua correlação já se justificaria por isso; tal correlação é reforçada especialmente por ser uma amostra exclusivamente de mulheres e esses serem os tipos de agressão sofrida mais comumente entre elas ^30^.

Em conjunto, os achados deste estudo sugerem que o InDI pode ser caracterizado por três medidas unidimensionais, cargas moderadas a fortes e cinco pares de itens possivelmente redundantes. Avaliações futuras do InDI devem se deter sobre as possíveis redundâncias identificadas neste trabalho. Para os pares com sobreposição mais evidente de conteúdo, como i3-i5 e i7-i8 (InDI-A), i11-i13 (InDI-D) e i28-i30 (InDI-M), recomenda-se sua combinação ou a exclusão de um dos itens da respectiva subescala. Quanto àqueles que não aparentam ter clara redundância de conteúdo (i.e., i17-18 do InDI-D), é possível que apresentem tal sobreposição especialmente entre mulheres no contexto do Brasil - não raro, perguntas inapropriadas e ofensivas são dirigidas a mulheres como estratégia para questionar sua inteligência ou capacidade. De qualquer forma, esses e outros pares de itens identificados como redundantes podem ser avaliados junto às populações-alvo do instrumento com entrevistas cognitivas ^31^, as quais permitiriam examinar se há de fato redundância ou se estamos diante de uma especificidade da amostra investigada.

Apesar de analisar amplamente as propriedades configurais e métricas do InDI, este estudo tem algumas limitações importantes, que devem ser consideradas. A amostra foi restrita a respondentes que se identificavam como mulheres cisgênero. Mesmo com o esforço de buscar uma amostra com maior diversidade, houve concentração nos estratos branco e heterossexual, residente nas regiões Sudeste e Sul do país, com alta escolarização; tal limitação implicou, potencialmente, menor variabilidade nas respostas aos itens, atenuando a magnitude das cargas fatoriais estimadas. Outras limitações referem-se à coleta de dados *online* e à divulgação da pesquisa via mídias sociais; ambas podem ter afetado as respostas aos itens em múltiplas direções, seja pelo acesso restrito à internet e mídias sociais por mulheres em situação de maior vulnerabilidade, pela divulgação acabar sendo limitada e se concentrar nas regiões Sul e Sudeste, ou ainda pela limitação de tempo e disponibilidade para responder uma pesquisa *online*. A administração *online* do questionário foi adotada devido à pandemia de COVID-19, que inviabilizou a coleta de dados presencial em instituições que atendem mulheres em situação de vulnerabilidade - plano original de seleção das participantes.

Os achados aqui documentados representam um passo adicional no processo de adaptação transcultural do InDI para o contexto brasileiro, utilizando uma amostra menos diversa do que aquela abordada nas etapas de versão do instrumento para o português ^20^. Neste trabalho, foram pesquisadas somente mulheres cisgênero, de diferentes orientações sexuais e cores/raças. Espera-se que esta pesquisa contribua para o desenvolvimento de outros trabalhos sobre interseccionalidade no campo da saúde, bem como para a redução das iniquidades em saúde por meio da maior visibilidade de grupos marginalizados e de suas experiências.

Em suma, as análises deste estudo apontaram para modelos unifatoriais com correlações residuais entre itens específicos. No InDI-A, a correlação foi entre itens relacionados à antecipação de discriminação financeira e à dificuldade em conseguir moradia, assim como à antecipação de agressões verbais e físicas. Já no InDI-D, os itens correlacionados abordam a percepção de o respondente ser uma pessoa agressiva e violenta, e como grupos minoritários são mais questionados e percebidos como menos capazes. No InDI-M, por sua vez, a correlação residual foi observada entre itens que abordam assédio físico e sexual. Recomenda-se que seja avaliada redundância dos itens com correlações residuais expressivas para exclusão de um do par ou a combinação de suas respostas quando das análises estatísticas. Além disso, estudos futuros devem examinar essas propriedades e a estrutura escalar do InDI para o português brasileiro em amostras mais amplas, bem como avaliar a invariância do instrumento entre grupos atravessados por gênero, classe social, idade, orientação sexual, cor/raça e suas intersecções.
